# News and Notes

**Published:** 1910-10

**Authors:** 


### NEWS AND NOTES

Dr. E. G. Ballinger is in Germany studying the technique of Erlich treatment for syphilis.

Dr. Louis M. Ganes has resigned as associate medical director of the Peachtree Heights Sanatorium.

Dr. Louis C. Rouglin formerly medical director and one of the founders of Pine Ridge Sanitarium has sold his interest in that institution, and is not now in connection with same. He has opened up his suite of offices at 747 Candler Annex, for general work, but is making a specialty of diseases of the lungs and chest.

Dr. C. C. Haskell, of the research department of the Lilly Laboratories, has returned to his office in Indianapolis after an absence of about three months. Dr. Haskell spent the summer studying infants' diseases in the hospitals of New York City.

### REGULAR MEETNG OF THE FULTON COUNTY MEDCAL SOCIETY.

### JULY 7TH., 1910.

REPORTED BY R. R. DALY, M. D.

---

In the Symposium on Pellagra, Dr. E. B. Block read a paper on "The Nervous Symptoms and Pathology," Dr. M. B. Hutchins read a paper on the "Skin and Mouth Symptoms" and Dr. LeConte read an essay on "The Digestive Symptoms."

Dr. Wolfe said in discussing it that the interest lies in diagnosis so far as the skin symptoms go. The three stages are not distinct, but are present for purposes of classification. The erythema is really a dermatitis; the desquametion varies from fine granules to large flakes, depending upon the amount of the inflammation, but atrophy varies in extent according to the injury of the skin and the time treatment is undertaken. The location is typical as a rule on the exposed surfaces but does not go all over invariably. Most of the cases resemble a fading eczema, but the glazed surface remaining is typical in its vc-iceous appearance. The palms of the hands show hyperkeratosis and look like a fowl's feet. This once seen, the diagnosis of the case is easy no matter what the variations. Sometimes in the lower limb there is ecchymosis of the watch-pocket appearance in places. Even in recovery the skin never gets perfectly clear. Pellagra sine pellagra may appear but it is hard to diagnosis. The tongue and mouth symptoms are not always synchronous with the skin signs. The intestinal and mental symptoms are more pronounced in adult life than in children. So far as the mental signs are concerned, the one he has noticed most often has been an explosion of anger; in others the mental dullness and confusion appear and when the tremor comes it is often general to the extent of causing the head to bob. Prostration is always great when it comes. The treatment is good nourishment and quiet rest in bed. The skin symptoms usually respond to simple measures if they are to be cured at all. The origin of the disease is hard to prove or disprove. Most of us in the South eat cornbread and evidence seems to be in favor of the maize ferment. In Central America there were two outbreaks of Pellagra after importation of corn from the United States following local famines.

Dr. Strickler said he was interested in Dr. LeConte's stomach findings. The earlier cases have an increase of free hydrochloric acid and marked absence of the acid later. None of us is in a position to say much as to the etiology; it is safer to follow the foreign writers who find the trouble due to corn and to advise our patients not to use this food. Where people live on good corn and are sure of it, no disease is to be discovered. He knows of a fatl case who never has eaten corn in good, but these statements must be taken cum grano salis. The eosinophiles in the blood was always increased though the intestinal parasites are rare. Pellagra does not occur before the skin symptoms appear. Cases of "incomplete sprue" have developed later in the pellagra with skin symptoms. Possibly the palm signs were there but he did not know of these at that time. In young people with sprue the palm always has the appearance of great age. His personal treatment has been unsuccessful. He is guided by the amount of acid in the stomach as to what foods he should give. As to drugs, Fowler's solution is the only one which seems to assist the patient in any way.

Dr. M. G. Campbell said that the method of handling and preparing corn and corn-meal was probably the cause of the trouble. Corn is not properly seasoned at the time it is gathered and after it is ground it is often placed in close grained sacks, causing heating and moulding.

Dr. Niles said that the diarrhoea in the beginning is compensatory and we should hesitate in checking it too soon. His finding of the stomach symptoms in eleven cases, except two, were below the normal acidity or entirely without acid. The diminishel tegumentary activity calls for other organs to do the skin's work. In the nervous symptoms there seems to be an actual change of the disposition so that men turn against their children and a "chronic grouch" often arises.

Dr. Lokey agreed with Dr. Campbell in the matter of the improper seasoning of the corn crops and he said that sometimes the ears are not sorted properly before the shelling process. He has seen hogs' tails drop off from being fed on smut corn.

Dr. Ward said that Dr. Campbell and Dr. Lokey were right as to the harvesting and handling of corn that is shipped in here.

Dr. LeConte said that he does not pretend to know that corn causes the trouble. If it does, some of the cases who say they never have eaten corn have had it in their adulterated white flour; it whitens wheat to have the corn added.

Dr. Block reviewed his statement with regard to the corn etiology. He does not know that corn is harmful but he keeps his patients from eating it. Of course, spoiled corn is bad for anybody. The poison is either a chemical one or a living organism. If there is a chemical poison, should it not resemble other chemical poisons in effect and the patient either get well or die and not have recurrent attacks? That there are recurrences shows that an organism does the work if it comes from corn at all; if due to mould, should we not find that mould in the bodies of the deceased? It never has been found and of course, it may be some other germ, but this never has been isolated although that is no proof that it does not exist. In Scarlet Fever and Measles the contagious organisms have never been isolated although we are reasonably sure of their presence. The same is likely to be true of Pellagra.

Dr. Goldsmith presented a case of a child five years old wearing twoartifiicial limbs. One amputation was at middle third of the thigh and one below the knee. He had been wearing them only 24 hours but could walk quite well. The injury occurred in a street car accident 2 years ago. The legs weigh 2 pounds 12 ounces. The case shows the advisability of early resort to artificial limbs.

Dr. Sutton presented a case of recovery from fracture of neck of femua which it had been necessary to wire together in the open method. There is practically no shortening though the man is adult. There had been failure of union after six weeks in a plaster cast.

